# Cross‐Vendor Validation of Proton Density Fat Fraction and T_1_ Mapping Using a Combined Proton Density Fat Fraction—T1 Phantom

**DOI:** 10.1002/jmri.70344

**Published:** 2026-05-20

**Authors:** Jitka Starekova, Sebastian Weingärtner, David R. Rutkowski, Garrett C. Fullerton, Won C. Bae, Hung P. Do, Ananth J. Madhuranthakam, Vadim Malis, Sheng Qing Lin, Suraj D. Serai, Takeshi Yokoo, Scott B. Reeder, Jean H. Brittain, Diego Hernando

**Affiliations:** ^1^ Department of Radiology University of Wisconsin‐Madison Madison Wisconsin USA; ^2^ Department of Imaging Physics Delft University of Technology Delft the Netherlands; ^3^ Calimetrix Madison Wisconsin USA; ^4^ Department of Medical Physics University of Wisconsin‐Madison Madison Wisconsin USA; ^5^ Department of Radiology University of California San Diego San Diego California USA; ^6^ Canon Medical Systems USA Tustin California USA; ^7^ Department of Radiology University of Texas, Southwestern Medical Center Dallas Texas USA; ^8^ Department of Radiology Mayo Clinic in Rochester Rochester Minnesota USA; ^9^ Department of Radiology, Children's Hospital of Philadelphia University of Pennsylvania School of Medicine Philadelphia Pennsylvania USA; ^10^ Department of Biomedical Engineering University of Wisconsin‐Madison Madison Wisconsin USA; ^11^ Department of Medicine University of Wisconsin‐Madison Madison Wisconsin USA; ^12^ Department of Emergency Medicine University of Wisconsin‐Madison Madison Wisconsin USA

**Keywords:** CSE‐MRI, PDFF, phantom, quantification, reproducibility, T_1_

## Abstract

**Background:**

In chronic liver disease, fat and fibroinflammatory changes often coexist. However, their biomarkers, proton density fat fraction (PDFF) and T_1_, are typically assessed separately. Their reproducibility under mutual confounding remains unclear.

**Purpose:**

To assess multicenter, multi‐vendor reproducibility of confounder‐corrected chemical shift‐encoded (CSE)‐MRI‐based PDFF mapping and MOLLI‐based T_1_ mapping using a combined PDFF‐T_1_ phantom.

**Study Type:**

Prospective phantom study.

**Phantom:**

Commercial PDFF‐T_1_ Phantom (Model 725) with varying PDFF (0%–30%) and T_1_ (200–1400 ms) values.

**Field Strength/Sequence:**

1.5 T and 3 T multi‐echo, three‐dimensional spoiled‐gradient‐echo (SGRE) sequence for PDFF mapping, and MOLLI sequence (5(3)3 acquisition scheme) using two‐dimensional SGRE readouts for T_1_ mapping across four centers and vendors.

**Assessment:**

PDFF and T_1_ maps were acquired using standardized protocols. PDFF maps were reconstructed locally, while T_1_ maps were generated using a centralized algorithm. All maps were quantitatively analyzed by a single radiologist using standardized region‐of‐interest placement. Phantom temporal stability was assessed at one center across five sessions over 9 months (baseline, retest, 1 week, 6 and 9 months).

**Statistical Tests:**

Intraclass correlation coefficients (ICC), reproducibility coefficients (RDC), and linear regression analysis were used. A *p* value < 0.05 was considered statistically significant.

**Results:**

PDFF showed overall excellent reproducibility (ICC = 0.987, RDC = 3.7%), with increased variability at higher T_1_ values (RDC up to 7.9% at T_1_ = 1400 ms). T_1_ mapping showed good reproducibility in the absence of fat (RDC 16–161 ms at PDFF = 0%), but moderate to poor reproducibility in the presence of fat, with RDC increasing up to 1553 ms at PDFF 30%. Temporal stability was excellent ICC ≥ 0.998 for both PDFF and T_1_, and RDC of 1.1%–1.3% for PDFF and 52–57 ms for T_1_.

**Data Conclusion:**

This phantom study demonstrated high reproducibility of PDFF, whereas T_1_ reproducibility deteriorated at higher fat and T_1_ levels, underscoring the need for fat‐corrected T_1_ mapping for reliable assessment of fibroinflammatory changes.

**Evidence Level:**

N/A.

**Technical Efficacy:**

Stage 1.

## Plain Language Summary

Magnetic resonance imaging (MRI) is used to measure liver fat and T1, a marker of tissue damage and inflammation. However, fat can interfere with T1 measurements. To test the reliability of these scans, we sent a specialized test object with known fat and T1 values to four hospitals. We found that while fat measurements were consistent across different MRI machines, T1 measurements varied significantly, especially when high levels of fat were present. This shows that standard MRI methods need improvement to ensure patients receive reliable liver disease assessments, regardless of which hospital or machine they visit.

## 1 Introduction

Metabolic dysfunction‐associated steatotic liver disease (MASLD) is the most prevalent chronic disease of the liver, affecting approximately 38% of the global adult population [1]. The earliest and hallmark feature of MASLD is excessive fat accumulation in the liver (steatosis). The presence of fat can progress to metabolic dysfunction‐associated steatohepatitis (MASH), a more severe form involving inflammation, hepatocellular injury, and fibrosis [1, 2]. If left untreated, the condition may advance to cirrhosis, liver failure, and hepatocellular carcinoma. MASLD is also linked to a range of extrahepatic complications [1, 3].

Given its high prevalence and progressive nature, there is an unmet need for reliable noninvasive biomarkers to assess liver fat and fibroinflammatory changes in both clinical practice and research settings [4, 5]. Confounder‐corrected chemical shift‐encoded MRI (CSE‐MRI) can measure proton density fat fraction (PDFF), a well‐validated quantitative biomarker for liver steatosis [3, 6]. T_1_ mapping has shown promise as a noninvasive biomarker for liver fibroinflammatory changes (via native T_1_) and function (via post‐contrast T_1_ using hepatocyte‐specific contrast agents) [7, 8].

However, both PDFF and T_1_ measurements can be affected by multiple confounding factors. The presence of fat leads to bias in T_1_ estimates when using conventional (fat‐uncorrected) methods. Changes in T_1_ values, such as those caused by fibroinflammatory changes, can lead to bias (overestimation) of PDFF estimates if no appropriate mitigation method, such as low flip angle or T_1_‐correction, is employed [3, 9, 10, 11, 12]. Reliable quantification of both steatosis and fibroinflammatory activity is important, as they have distinct implications for MASLD diagnosis, staging, and treatment strategies in the clinic.

Chemical shift encoded MRI (CSE‐MRI)‐based PDFF quantification is considered the leading noninvasive assessment method for quantifying liver steatosis [3]. Among existing T_1_ mapping techniques, modified Look‐Locker inversion recovery (MOLLI) methods, originally developed for cardiac imaging, are widely available and have been used for liver imaging [13, 14]. However, MOLLI is susceptible to bias in the presence of fat [13]. Given the high prevalence of steatosis, the reproducibility of MOLLI‐based T_1_ mapping in the clinical setting remains uncertain.

Multicenter studies enable highly controlled validation of quantitative MRI methods across centers, vendors, and platforms. PDFF and T_1_ phantoms have been used in several multicenter studies [15, 16, 17, 18, 19, 20]. However, there is limited data on the reproducibility of PDFF and T_1_ mapping in the simultaneous presence of *varying combinations of PDFF and T*_*1*_ *values*.

Therefore, this study evaluated the multicenter, multi‐vendor reproducibility of CSE‐MRI‐based PDFF and MOLLI‐based T_1_ quantification at 1.5 T and 3.0 T using a combined quantitative PDFF‐T_1_ phantom.

## 2 Materials and Methods

This prospective phantom study was conducted across four participating centers (University of Wisconsin–Madison, University of Texas Southwestern Medical Center, Children's Hospital of Philadelphia, and Canon Medical Systems USA's Training Academy, Irvine, California) from February 2022 to December 2022.

### 2.1 PDFF‐T_1_ Phantom

**FIGURE 1:**
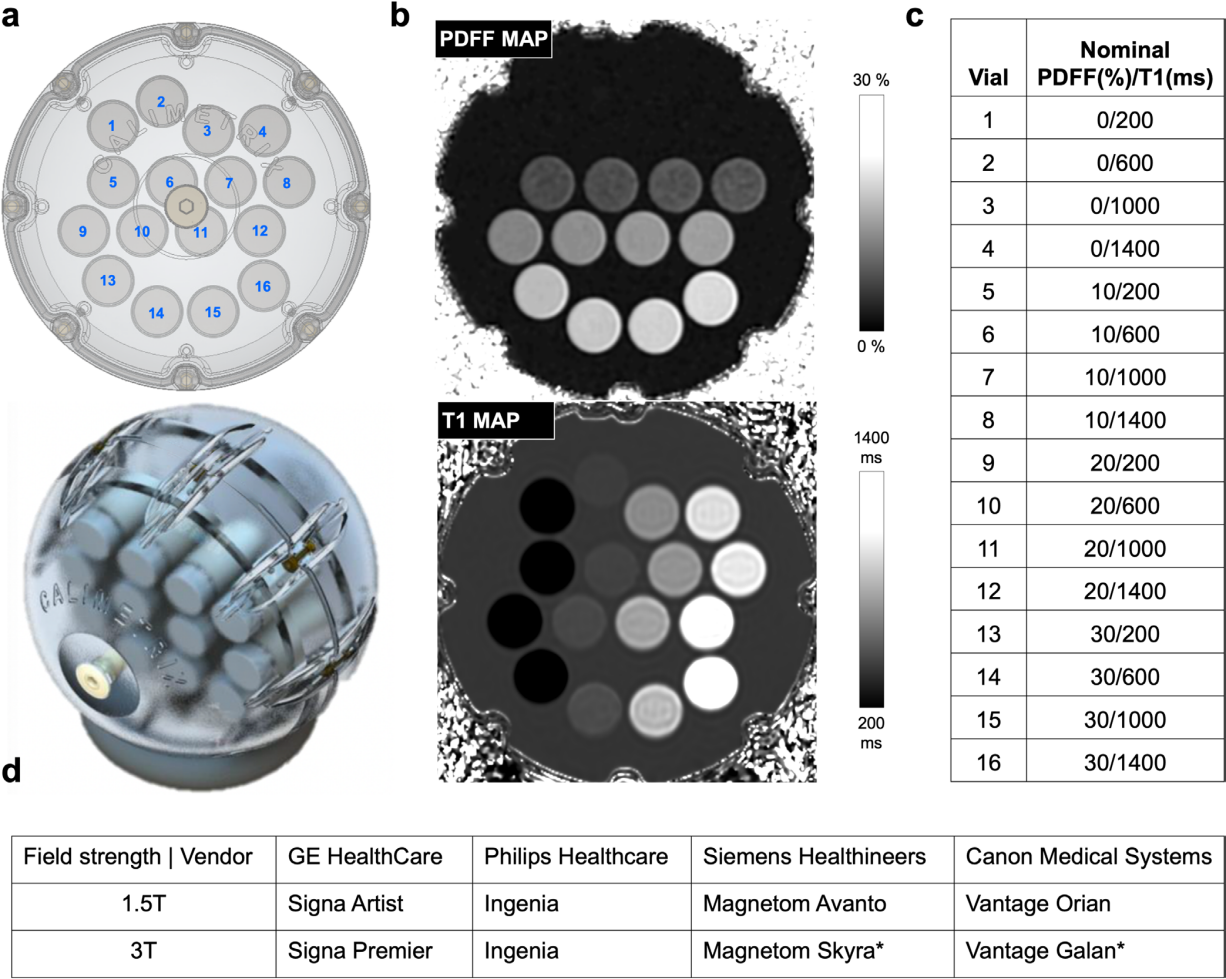
Illustration and photograph of the PDFF‐T_1_ phantom (a) with corresponding PDFF and T_1_ map (b). The phantom was designed as an array of 16 vials immersed in a doped fill solution, contained within a spherical housing. Each vial contained a distinct combination of PDFF and water‐specific T_1_ values (nominal values) as shown in the table (c). The phantom was imaged at four different centers with different MR vendors (d). Abbreviation: PDFF, proton density fat fraction. *2.89 T.

A single commercial phantom (Model 725 Special Order PDFF‐T_1_ Phantom, Calimetrix, Madison, WI) was used in this study. The phantom includes 16 cylindrical vials (volume: 20 mL, length: 60 mm, outer diameter: 27 mm) arranged in an asymmetrical grid. Each vial was filled with a gel formulation designed to mimic a specific combination of nominal PDFF values (0%–30%) and nominal water‐specific T_1_ (200–1400 ms), as specified by the phantom manufacturer (Figure 1). These values were selected to span the physiologically relevant range of PDFF and water‐specific T_1_ values observed in the human liver, including both native and post‐contrast conditions [6]. The phantom gels achieve water‐specific T_1_ values that remain approximately the same at 1.5 T and 3.0 T. To improve magnetic field homogeneity, coil loading, and minimize artifacts, the phantom's spherical housing was filled with a doped water solution. Water solution doping shortens the T_1_ and T_2_ relaxation times to better match the background contrast of human tissue. Additionally, gel‐based vials were used to reduce motion‐related and susceptibility artifacts caused by air bubbles.

Reference PDFF values for each vial were defined by phantom construction and verified through measurements using a temperature‐adjusted, confounder‐corrected reconstruction of CSE‐MRI data [21]. T_1_ values in the fat‐free vials were verified using a conventional inversion‐recovery spin‐echo sequence (flip angle = 90°, TE = 9 ms, TR = 7000 ms, TI = 50, 400, 900, 1800 ms). T_1_ estimates were obtained using magnitude‐based exponential fitting across inversion times. Verification imaging at 3.0 T yielded values of 195 ± 1.7 ms, 619 ± 3.8 ms, 1025 ± 16.4 ms, and 1435 ± 19.6 ms. For vials with fat content, water‐specific T_1_ was assumed to be the same as in the absence of fat (i.e., the nominal water‐specific T_1_ values were assumed to be constant across PDFF values) [10]. Vial verification was performed in accordance with standard Calimetrix Quality Management System (QMS) procedures.

### 2.2 Imaging Experiments

The study involved four centers, each equipped with MRI systems from a different vendor (Figure 1). Specifically, the following vendors and systems were included: GE HealthCare 1.5 T Signa Artist and 3.0 T Signa Premier (Waukesha, WI, USA) at University of Wisconsin–Madison (center 1); Philips Healthcare 1.5 T and 3.0 T Ingenia systems (Amsterdam, Netherlands) at University of Texas Southwestern Medical Center (center 2); Siemens Healthineers 1.5 T Magnetom Avanto and 2.89 T Magnetom Skyra (Erlangen, Germany) at Children's Hospital of Philadelphia (center 3); and Canon Medical Systems 1.5 T Vantage Orian and 2.89 T Vantage Galan (Otawara, Tochigi, Japan) at Canon Medical Systems USA's Training Academy, Irvine, California (center 4).

Two quantitative techniques were employed [1]: a 3D multi‐echo, spoiled‐gradient‐echo (SGRE) sequence for PDFF mapping, and [2] a MOLLI sequence with 5(3)3 acquisition scheme using 2D SGRE readouts for T_1_ mapping. A low flip angle SGRE readout was used with MOLLI to minimize fat‐ and B_0_ inhomogeneity‐related effects on T_1_ estimates [22]. Attempts were made to match acquisition parameters across centers and vendors; however, exact alignment was constrained by hardware and pulse sequence variations among vendors. Of note, T1 mapping could not be performed on the Philips 1.5 T system at Center 2 as the SGRE‐based MOLLI sequence was unavailable on that specific platform.

For PDFF quantification, centers were instructed to match the following acquisition parameters: number of slices 14, slice thickness of 4 mm, TE_1_ of 1.0–1.2 ms (for both 1.5 T and 3 T MRI systems), ΔTE of 1.8–2.1 ms for 1.5 T and 0.8–1.0 ms for 3 T, six echoes acquired in a single echo train for 1.5 T and two interleaved echo trains for 3 T, and a flip angle of 3 for both 1.5 T and 3 T (Table 1).

**TABLE 1:** Imaging parameters for proton density fat fraction (PDFF) mapping acquisition.

Parameter	Recommended	Center 1 GE HealthCare	Center 2 Philips Healthcare	Center 3 Siemens Healthineers	Center 4 Canon Medical Systems
1.5 T					
Coil/channels (n)	Head or head–neck	Head–neck/21	Head/15	Head/8	Head–neck/16
Matrix size	148 × 148	148 × 148	144 × 144	128 × 128	144 × 144
FOV (cm^2^)	26 × 26	26 × 26	26 × 26	26 × 26	26 × 26
Slice thickness (mm)	4	4	4	4	4
First TE (ms)	1.0 to 1.2	1.1	1.2	1.11	1.2
TE spacing (ms)	1.8 to 2.1	1.9	1.3	2.0	1.2
TR (ms)	Min^a^	23.0	8.9	12.5	8.8
No. of echo trains/echoes	1/6	1/6	1/6	1/6	1/6
FA (degrees)	3	3	5	3	3
Pixel BW (Hz)	1351	1351	1578	1085	1302
3 T^c^					
Coil/channels (n)	Head or head–neck	Head–neck/19	Head/32	Head/8	Head–neck/16
Matrix size	140–160 × 140–160	160 × 160	164 × 163	128 × 128	144 × 144
FOV (cm^2^)	26 × 26	26 × 26	26 × 26	26 × 26	26 × 26
Slice thickness (mm)	4	4	4	4	4
First TE (ms)	1.0‐1.2	1.1	1.2	1.11	1.2
TE spacing (ms)	0.8 to 1.0	0.9	1.0	2.0	1.0
TR (ms)	Min^a^	7.3	7.5	12.5	7.6
No. of echo trains/echoes	2/6	2/6	1/6	1/6^b^	1/6
FA (degrees)	3	3	3	3	3
Pixel BW (Hz)	1136 to 1429	1136	1494	1090	1302

Abbreviations: BW, bandwidth; FA, flip angle; FOV, field of view; No, number; TE, echo time; TR, repetition time.

^a^ Minimum achievable with the provided echo times and other system constraints.

^b^ Bipolar readout.

^c^ 2.89 T for Siemens and Canon.

For T_1_ quantification, centers were instructed to use the following acquisition parameters: number of slices 1 through the middle of the vials, slice thickness of 4 mm, TI_1_ of 97–137 ms for 1.5 T and 97–115 ms for 3 T MRI systems, ΔTI of 80 ms for both 1.5 T and 3 T, TR of 2.8–3.7 ms for both 1.5 T and 3 T, flip angle of 12° for 1.5 T and 10° for 3 T, 5(3)3 acquisition scheme (Table 2).

**TABLE 2:** Imaging parameters for T_1_ mapping acquisition.

Parameter	Recommended	Center 1 GE HealthCare	Center 2 Philips Healthcare	Center 3 Siemens Healthineers	Center 4 Canon Medical Systems
1.5 T					
Coil/channels (n)	Head or head–neck	Head–neck/21	n/a	Head/8	Head–neck/16
FOV (cm^2^)	26 to 36	36 × 27		26 × 21	36 × 27
Matrix size	128 × 128	128 × 128		128 × 128	128 × 128
Slice thickness (mm)	6 to 8	8		8	6
TR (ms)	2.8 to 3.7	3.1		3.6	3.6
Initial TI (ms)	97 to 137	98		100	97
ΔTI (ms)	80	80		100	80
Pixel BW (Hz)	480–980	488		1090	977
FA (degrees)	12	12		12	12
Acquisition scheme	5(3)3	5(3)3		5(3)3	5(3)3
3 T^a^					
Coil/channels (n)	Head or head–neck	Head–neck/21	Head/15	Head/8	Head–neck/16
FOV (cm^2^)	26 to 36	36 × 27	30 × 24	26 × 21	36 × 27
Matrix size	128 × 128	128 × 128	132 × 126	128 × 128	128 × 128
Slice thickness (mm)	6 to 8	8	8	8	8
TR (ms)	2.8 to 3.7	3.1	3.0	3.7	3.3
Initial TI (ms)	97 to 115	103	85	100	97
ΔTI (ms)	80	80	80	100	80
Pixel BW (Hz/pixel)	480–980	488	553	781	977
FA (degrees)	10	10	10	10	10
Acquisition scheme	5(3)3	5(3)3	5(3)3	5(3)3	5(3)3

Abbreviations: BW, bandwidth; FA, flip angle; FOV, field of view; T_1_, inversion time; TR, repetition time.

^a^ 2.89 T for Siemens and Canon.

The phantom was shipped to participating centers via an overnight courier service in a protective, foam‐padded case to reduce the risk of damage during transit (Figure S1). All centers were instructed to store the phantom at room temperature when not in use and to allow it to acclimate to the MRI room temperature for at least 30 min before data acquisition, to ensure optimal image quality and consistency. The phantom was leveled within the head or head–neck coil using foam pads and a manufacturer‐provided rubber base (where compatible) to accommodate varying coil geometries and secure the phantom to prevent movement during data acquisition. Reproducible positioning was ensured by aligning the system's laser crosshairs with the phantom's integrated orientation markings (cross) to place the phantom at the magnet isocenter.

The temperature of the phantom was measured just before imaging using a self‐adhesive temperature‐indicating strip (Cole‐Parmer, Vernon Hills, IL, USA). Upon return to Center 1 after multicenter imaging, the phantom housing and vials were visually inspected by the study coordinator to ensure no damage had occurred during transit prior to stability testing.

To evaluate the stability of the phantom's PDFF and T_1_ values over time, multiple repeated acquisitions were performed at Center 1 using 1.5 T and 3.0 T systems above, following the same imaging protocols described above using the same hardware and software. Two exams were conducted during the initial session on the same day: a baseline test and a retest exam performed following repositioning of the phantom and reconnection of the coil. Follow‐up exams were conducted at 1 week, 6 months, and 9 months (Figure S1).

### 2.3 Image Processing and Quantitative Analysis

PDFF maps were automatically reconstructed online at the respective centers using the vendor‐provided reconstruction software and subsequently transferred to Center 1 for analysis. Because vendor‐specific T_1_ map reconstruction was not available for some systems, all T_1_ maps from the participating centers were processed centrally at Center 1 using a standardized MOLLI fitting algorithm implemented in Matlab (MathWorks, Natick, MA) [23, 24].

An experienced radiologist (16 years of MRI experience, JS) used OsiriX DICOM viewer (v14.1.2 Pixmeo, Bernex, Geneva, Switzerland) to place circular regions of interest (ROIs), 1.9 cm in diameter, on each of the 16 vials per slice. For the PDFF maps, ROIs were placed on three consecutive central slices, and voxel values were averaged across them. For the T_1_ map, ROIs were placed on a single central slice, and the mean voxel value was recorded.

### 2.4 Validation of Phantom Homogeneity

To verify the spatial homogeneity of the phantom and provide an independent reference for water‐specific T_1_ in the presence of fat, an additional validation was performed at Center 1 (3.0 T). A saturation‐recovery chemical shift‐encoded (SR‐CSE) method was used to achieve simultaneous fat‐water separation and T_1_ mapping of the water compartment (water‐specific T_1_) [25]. Acquisition parameters included: FOV 28 × 28cm^2^, 128 × 96 matrix, 2 × auto‐calibrated parallel imaging in the phase‐encoding direction, 8.59 ms TR, 5 monopolar echoes per TR (TE1 = 1.24 ms, ∆TE = 1.41 ms), 3 saturation‐recovery preparations (TS = [200 ms, 715 ms, and 1231 ms]) and one acquisition without saturation preparation, 4 mm slice thickness, 6 mm gap between adjacent slices, averaged across 5 NEX. To assess phantom homogeneity, standardized ROIs were drawn in three spatially separated slices of water‐specific T1 maps along the length of each vial. Mean and standard deviation of water‐specific T1 measurements were computed for each vial across the three slices.

### 2.5 Statistical Analysis

Statistical analysis was performed using R (v4.1.0., tidyverse v1.3.1, ggplot2 v.3.3.6, irr v.0.84.1, rstatix 0.7.0). For the multicenter, multi‐vendor validation of PDFF and T_1_ mapping, the intraclass correlation coefficients (ICC) were calculated to assess overall agreement across scanners and vendors. ICC values were interpreted as < 0.50 = poor, 0.50–0.75 = moderate, 0.75–0.90 = good, and > 0.90 = excellent [26]. Reproducibility coefficients (RDC) were calculated to quantify measurement precision. Overall RDC values were calculated by pooling data across all vials within specific nominal ranges (PDFF: 0%–30%; T_1_:200–1400 ms) to provide a global estimate of measurement variation. Linear regression was performed using measured PDFF/T_1_ values as the dependent variables and corresponding nominal phantom values as the independent variables to quantify linear association. To assess measurement consistency over time (i.e., phantom stability), the ICC and RDC were similarly calculated. For the validation of phantom homogeneity, the mean, standard deviation, coefficient of variation (CV), and ICC were calculated.

Outlier detection was performed using the interquartile range (IQR) method. Data points were considered outliers if they fell below the first quartile or above the third quartile by more than 1.5 times the IQR and classified as extreme outliers if they exceeded three times the IQR.

For all tests, *p* < 0.05 was considered statistically significant.

## 3 Results

PDFF and T_1_ mapping data were successfully acquired at all centers except for T1 mapping at Center 2 (1.5 T Philips). The measured phantom temperature prior to imaging ranged from 18°C to 23°C across all centers and systems.

### 3.1 PDFF Reproducibility

**FIGURE 2:**
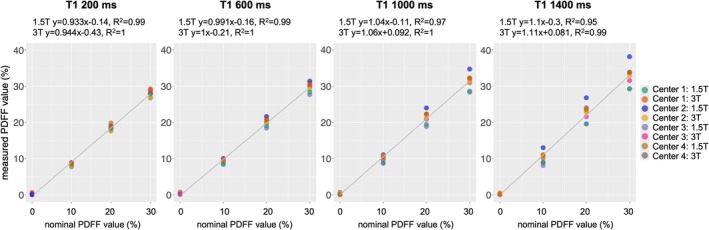
Linear regression shows good agreement in PDFF values across centers and platforms, with higher variability in the presence of high fat and T_1_. Depicted are reference (nominal) and measured CSE‐MRI PDFF values across 4 centers and 8 different systems. The columns represent vials with nominal water‐specific T_1_ of 200 ms, 660 ms, 1000 ms and 1400 ms, respectively. The regression equations predict measured PDFF (y‐axis) based on nominal PDFF (x‐axis). Vendors: Center 1, GE HealthCare; Center 2, Philips Healthcare; Center 3, Siemens Healthineers; Center 4, Canon Medical Systems. Abbreviations: PDFF, proton density fat fraction; CSE‐MRI, chemical shift‐encoded MRI.

Overall, excellent agreement was observed for PDFF measurements across centers and vendors (ICC 0.987 [95% CI: 0.975–0.995], RDC 3.7%, Table 3). Linear regression analysis demonstrated a strong linear association between measurements (R^2^ range: 0.95–1.00, Figure 2).

**TABLE 3:** Reproducibility coefficient for chemical shift‐encoded proton density fat fraction (PDFF) and low flip angle spoiled‐gradient‐echo‐based MOLLI T_1_ mapping.

PDFF Overall RDC: 3.7%					T_1_ Overall RDC: 499 ms				
Vial	PDFF (%)	T_1_w (ms)	SD	RDC (%)	Vial	PDFF (%)	T_1_w (ms)	SD	RDC (ms)
1	0	200	0.25	0.71	1	0	200	5.6	15.6
2	0	600	0.28	0.77	2	0	600	13.1	36.3
3	0	1000	0.23	0.63	3	0	1000	28.5	79.1
4	0	1400	0.23	0.63	4	0	1400	58.2	161.4
5	10	200	0.45	1.25	5	10	200	9.0	24.9
6	10	600	0.52	1.44	6	10	600	22.9	63.6
7	10	1000	0.88	2.43	7	10	1000	47.0	130.4
8	10	1400	1.57	4.34	8	10	1400	86.3	239.1
9	20	200	0.76	2.09	9	20	200	7.8	21.7
10	20	600	1.02	2.84	10	20	600	44.2	122.4
11	20	1000	1.69	4.68	11	20	1000	131.8	365.4
12	20	1400	2.41	6.68	12	20	1400	298.3	826.7
13	30	200	0.89	2.48	13	30	200	8.7	24.2
14	30	600	1.25	3.46	14	30	600	90.4	250.6
15	30	1000	2.09	5.78	15	30	1000	269.9	748.1
16	30	1400	2.86	7.93	16	30	1400	560.1	1552.5

Abbreviation: RDC, Reproducibility coefficient.

Some variability was observed at low T_1_ values (RDC 0.7%–3.5% for T_1_ ≤ 600 ms), whereas higher variability occurred at longer T_1_ (RDC up to 7.9% for T_1_ = 1400 ms and PDFF 30%) (Table 3, Figure 2, and Figure S2).

### 3.2 T_1_ Reproducibility

**FIGURE 3:**
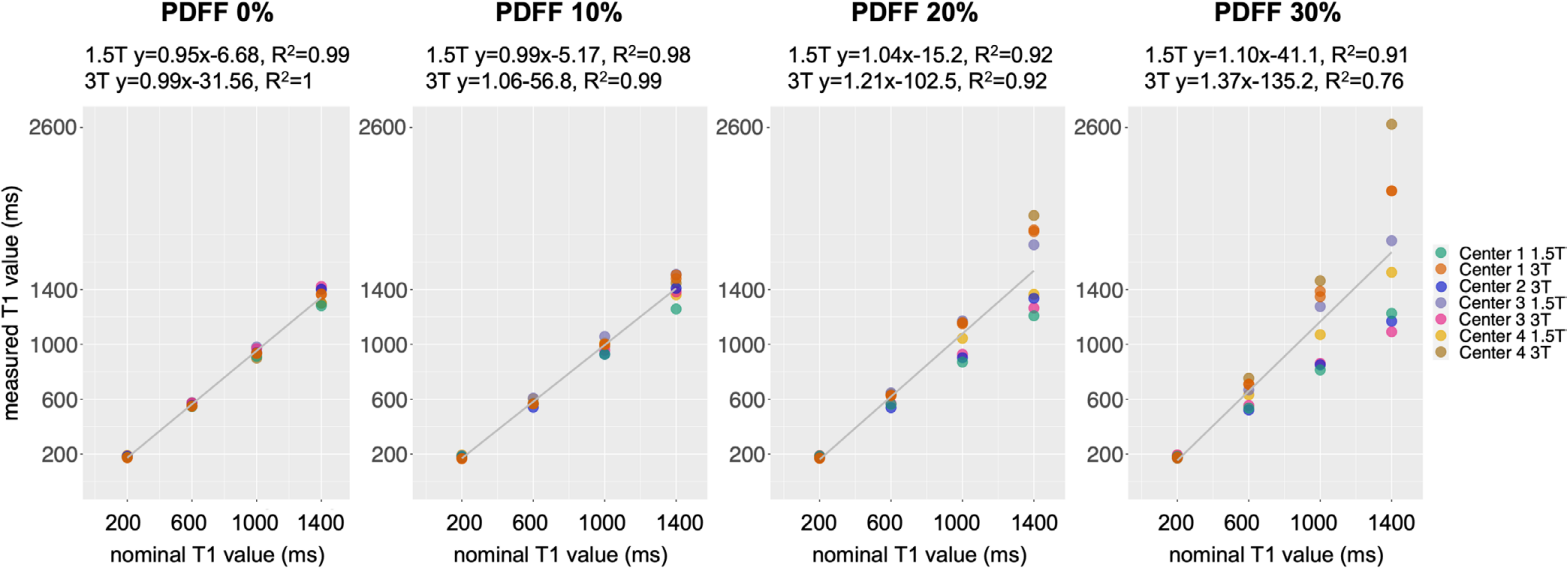
Linear regression shows observed agreement in T_1_ values across the centers and platforms, with low variability in the absence of fat, and higher variability in the presence of high fat and T_1_. Depicted are nominal water‐specific T_1_ values and measured T_1_ values from low flip angle SGRE T_1_ mapping across 4 centers and 7 different scanners. Unlike in vivo, where T_1_ values vary depending on the scanner's field strength, T_1_ values in the phantom are nearly the same at 1.5 T and 3 T. The regression equations predict measured T_1_ (y‐axis) based on nominal T_1_ (x‐axis). Vendors: Center 1, GE HealthCare; Center 2, Philips Healthcare; Center 3, Siemens Healthineers; Center 4, Canon Medical Systems. Abbreviation: SGRE, spoiled‐gradient‐echo.

In fat‐free vials (PDFF 0%), excellent agreement was observed for MOLLI‐based T_1_ measurements across centers and vendors (ICC 0.996 [95% CI 0.984–1.0]). Across all vials, including those with fat, overall agreement was good (ICC 0.885 [95% CI 0.793–0.951], RDC 499 ms, Figure 3). Linear regression analysis showed moderate linear association between measurements (R^2^ range: 0.76–1; Figure 3).

Variability increased with higher PDFF and longer T_1_, with RDC values ranging from 16 ms (Vial 1, 0% PDFF) to 1553 ms (Vial 16, 30% PDFF) (Table 3, Figure 3).

### 3.3 Phantom Temporal Stability

**FIGURE 4:**
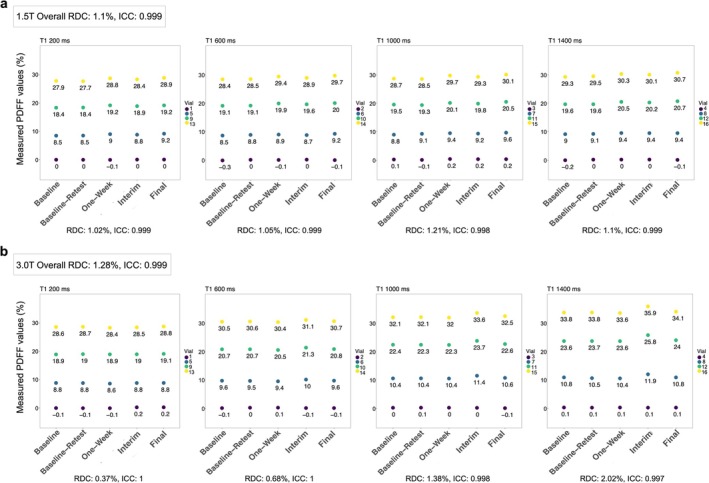
The phantom was imaged at Center 1 several times to test its stability, before and after it was returned to Center 1. Excellent agreement was observed for PDFF between these acquisitions with an overall ICC = 0.999 at 3.0 T and 1.5 T. Two exams were performed during the initial session (same day): A baseline exam and a retest after repositioning the phantom and reconnecting of the coil. Follow‐up exams were conducted at 1 week, 6 months (interim exam), and 9 months later (final exam). Abbreviation: RDC, reproducibility coefficient.

Upon return to Center 1, inspection of the phantom housing and vials revealed no visible signs of damage. Excellent agreement was observed between initial and follow‐up acquisition measurements at Center 1 for PDFF, with an overall ICC of 0.999 (1.1%) at 1.5 T, and ICC of 0.999 (RDC 1.28%) at 3.0 T (Figure 4).

**FIGURE 5:**
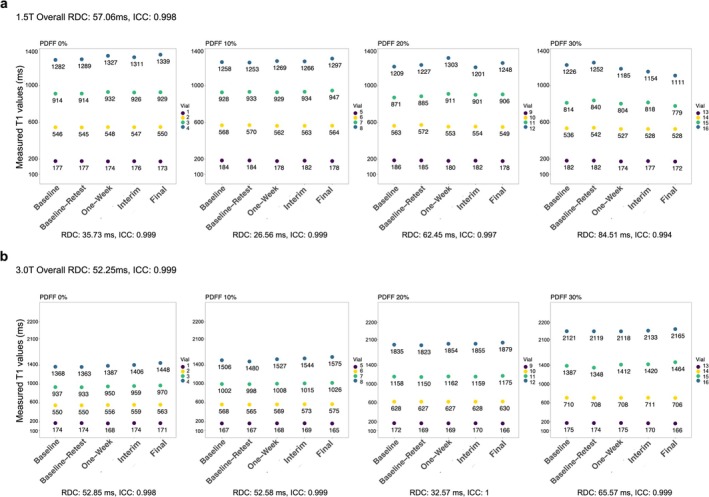
The phantom was imaged at Center 1 several times to test its stability, before and after it was returned to Center 1. Excellent agreement was observed (ICC = 0.999 at 3.0 T, and ICC = 0.998 at 1.5 T). A slight upward drift in T_1_ values was noted at 3.0 T but not at 1.5 T. Two exams were performed during the initial session (same day): A baseline exam and a retest after repositioning the phantom and reconnecting of the coil. Follow‐up exams were conducted at 1 week, 6 months (interim exam) and 9 months later (final exam). Abbreviation: RDC, reproducibility coefficient.

Similarly, excellent agreement was observed between initial and follow‐up acquisition measurements for T_1_ values at Center 1 (Figure 5), with an overall ICC of 0.999 (RDC 52 ms) at 3.0 T, and an ICC of 0.998 (RDC 57 ms) at 1.5 T. While a slight trend of increasing T_1_ values over time was observed at 3.0 T (e.g., a maximal increase of 80 ms [5.8%] in vial 4 over 9 months), this trend was not apparent on the 1.5 T data.

The surface temperature of the phantom, as indicated by the temperature‐indicating strip before each imaging session, ranged between 19°C–20°C at 3.0 T and 18°C–20°C at 1.5 T.

### 3.4 Validation of Phantom Homogeneity

The SR‐CSE validation confirmed high spatial homogeneity across all phantom vials. The CV for T1 measurements across spatially separated slices ranged from 0.02% to 2.43%, with an ICC of 1.0 [95% CI: 0.999–1.0], indicating negligible through‐plane variation (detailed results provided in Table S2).

## 4 Discussion

In this work, we conducted a phantom study across four centers with four vendors and multiple platforms at both 1.5 T and 3 T, using CSE‐MRI and MOLLI‐based protocols to evaluate the reproducibility of PDFF and T_1_ quantification, respectively. Since abnormal fat accumulation and fibroinflammatory changes often coexist in liver diseases such as MASLD, joint evaluation of both biomarkers has high clinical relevance.

Using a commercially available PDFF‐T_1_ phantom with concomitantly modulated PDFF and T_1_ values, we demonstrated overall high multicenter, multi‐vendor reproducibility of CSE‐MRI based fat quantification at both 1.5 T and 3 T. Agreement was excellent across most tested PDFF‐T_1_ combinations; however, reproducibility deteriorated at higher T_1_ values and higher PDFF levels, with increased variability and systematic overestimation of PDFF. These findings indicate that while PDFF measurements are robust across a broad physiological range, measurement precision is reduced under conditions of prolonged T_1_ and high fat content, which are clinically relevant in advanced liver disease.

This behavior is consistent with the known limitations of conventional PDFF mapping methods, which may exhibit residual T_1_‐bias depending on the T_1_‐weighting parameters (TR, flip angle) and the differences between the T_1_ value of water and the T_1_ value of fat [11, 27]. Such bias becomes increasingly relevant in tissues with prolonged water T_1_, including those affected by fibroinflammatory changes, and may therefore impact PDFF quantification in progressive stages of MASLD [27]. Further mitigation of T_1_ bias is likely achievable through post‐processing approaches (given the known acquired flip angles and assumed or measured T_1_ values), or by employing nonsteady state T_1_‐independent acquisitions [28, 29, 30].

Our findings highlight the need for the development of confounder‐corrected techniques to ensure accurate and reliable T_1_ quantification across sites and patient populations. While MOLLI‐based T_1_ mapping is reproducible at low PDFF, it is confounded at higher PDFF values due to fat‐related signal interference, resulting in substantial bias and variability in T_1_ measurements [13]. Standardization strategies, such as system calibration and consistent acquisition protocol, may partially mitigate these limitations. Alternatively, several confounder‐corrected liver T_1_ mapping techniques have been proposed, correcting for the presence of fat [9, 10, 13, 25, 31, 32]. Once such advanced T_1_ mapping methods are implemented across all major vendors, multicenter phantom studies will be needed to validate and benchmark performance. Confounder‐corrected methods may enable increased reproducibility of T_1_ mapping of the liver, even in the presence of elevated PDFF.

Excellent longitudinal stability of T_1_ values was observed at 1.5 T. A slight upward trend in T_1_ values was noted at 3.0 T. This upward trend at 3.0 T may be due to confounding factors that impact MOLLI‐based T_1_ quantification, including B_0_ drift, shimming, or RF instability. Drift caused by temperature fluctuation appears less likely given the lack of monotonic changes in temperature across longitudinal scans.

## 5 Limitations

Although the phantom was temperature‐stabilized prior to scanning at each site, maintaining uniform temperature across sites and systems in the MRI suite was not possible and may have introduced variability between sites. Temperature fluctuations during imaging due to system heating may partially explain discrepancies between the estimated and reference PDFF and T_1_ values. However, our acquisition protocol represents a realistic scenario for real‐world deployment of phantom‐based quality assurance. Therefore, this slight variability is likely representative of the subsequent use of phantom‐based quality assurance in the clinic or in clinical trials. Furthermore, any temperature dependence may vary across vendors for PDFF due to differences in reconstruction algorithms. In contrast, T_1_ maps were reconstructed offline using a single method (as some systems lacked online reconstructions for the acquisition parameters used in this project), minimizing such variability [16].

Another aspect to consider is the use of a head or head–neck coil rather than a body array coil, which would typically be used in clinical abdominal imaging. While this choice was made to facilitate consistent and reproducible positioning across centers, it may affect image quality parameters such as signal‐to‐noise ratio and coverage. While B1 inhomogeneity is a known confounder for MOLLI, its impact was minimized by the small size of the phantom and position at isocenter. While important, this effect was considered secondary to the more pronounced confounding effects of fat observed in this study.

Our study was based exclusively on phantoms rather than ex vivo or in vivo tissue. While this limits the direct clinical applicability of our findings, it provides greater control over imaging conditions. Conducting a study involving patient or specimen imaging across multiple centers would be logistically challenging [33]. Finally, although we included four major MRI vendors to enhance generalizability, expanding to additional platforms and clinical environments would further strengthen the applicability of our results.

## 6 Conclusion

This multicenter, multi‐vendor, multi‐platform study demonstrated high reproducibility of CSE‐based PDFF measurements, using a phantom with simultaneously modulated PDFF and T_1_ values. Although PDFF measurements were generally robust, reproducibility deteriorated under conditions of prolonged T_1_ and high fat content, which are common in advanced liver disease. In contrast, MOLLI‐based T_1_ mapping showed markedly reduced reproducibility in the presence of increasing fat content, with substantial variability at higher PDFF levels. These findings support the established high reproducibility of PDFF as a quantitative imaging biomarker and emphasize the importance of further development and validation of T_1_ mapping techniques, particularly for use in patients with coexisting steatosis and fibroinflammatory changes. Our results support the development and standardization of liver MRI biomarkers across clinical trials and clinical practice.

## Funding

J.H.B. and D.H. report grant support from: NIH R41 EB025729, NIH R44 EB025729. S.B.R. and D.H. report grant support from: NIH R01 EB031886. A.J.M. reports grant support from: NIH R01 CA283663. S.W. acknowledges funding from: European Research Council ERC Grant no. 101078711; Nederlandse Hartstichting, Grant no. 03‐004‐2022‐0079.

## Disclosure

S.B.R. has ownership interests in Calimetrix, Madison, WI, USA. Unrelated to this work, he reports ownership interests in Reveal Pharmaceuticals, VistaAI, RevOps, Elucent Medical, and he provides consulting services for Amgen. He receives research support from the John H. Juhl Professorship. The University of Wisconsin‐Madison received research support from GE HealthCare and Bracco Diagnostics.

## Supporting information


**Figure S1:** Phantom shipping workflow and imaging schedule across centers imaging at Center 1 was performed at multiple points throughout the study to evaluate phantom integrity and monitor potential drift in proton density fat fraction (PDFF) and T_1_ values. Two exams were conducted during the initial session on the same day (a baseline exam and followed by a retest after repositioning the phantom and reconnecting the coil). Follow‐up exams took place at 1 week, 6 months (interim exam; after the phantom was shipped from Center 3 to Center 1) and 9 months (final exam). The phantom was transported between participating centers using an overnight courier service within a protective, foam‐padded case.
**Figure S2:** Good reproducibility with low bias was observed for PDFF mapping across PDFF values (0%–30%) and T_1_ values (200–1400 ms). A moderate overestimation and increased variability were observed in measurements with long T_1_, particularly at higher PDFF values. This effect is likely due to residual T_1_ weighting in conventional PDFF mapping methods. These methods use a low flip angle between 3°C–5°C, which still leads to residual T_1_ bias when the T_1_ of water is much longer than the T_1_ of fat (approximately 350 ms).
Note: PDFF, proton density fat fraction.
**Table S1:** Proton density fat fraction (PDFF) and T_1_ reproducibility coefficient (RDC) and percentage RDC (% RDC).
**Table S2:** Validation of phantom homogeneity via saturation‐recovery chemical shift‐encoded (SR‐CSE) T_1_ mapping.
