# Supplementary material for: Cross‐Vendor Validation of Proton Density Fat Fraction and T1 Mapping Using a Combined Proton Density Fat Fraction—T1 Phantom
Source: J Magn Reson Imaging. 2026 May 20;64(2):482–92. doi: 10.1002/jmri.70344 (PMC13356357; doi:10.1002/jmri.70344)
Supplement: Supplementary file 1 — Figure S1: Phantom shipping workflow and imaging schedule across centers imaging at Center 1 was performed at multiple points throughout the study to evaluate phantom integrity and monitor potential drift in proton density fat fraction (PDFF) and T1 values. Two exams were conducted during the initial session on the same day (a baseline exam and followed by a retest after repositioning the phantom and reconnecting the coil). Follow‐up exams took place at 1 week, 6 months (interim exam; after the phantom was shipped from Center 3 to Center 1) and 9 months (final exam). The phantom was transported between participating centers using an overnight courier service within a protective, foam‐padded case. Figure S2: Good reproducibility with low bias was observed for PDFF mapping across PDFF values (0%–30%) and T1 values (200–1400 ms). A moderate overestimation and increased variability were observed in measurements with long T1, particularly at higher PDFF values. This effect is likely due to residual T1 weighting in conventional PDFF mapping methods. These methods use a low flip angle between 3°C–5°C, which still leads to residual T1 bias when the T1 of water is much longer than the T1 of fat (approximately 350 ms). Note: PDFF, proton density fat fraction. Table S1: Proton density fat fraction (PDFF) and T1 reproducibility coefficient (RDC) and percentage RDC (% RDC). Table S2: Validation of phantom homogeneity via saturation‐recovery chemical shift‐encoded (SR‐CSE) T1 mapping. [file JMRI-64-482-s001.docx]

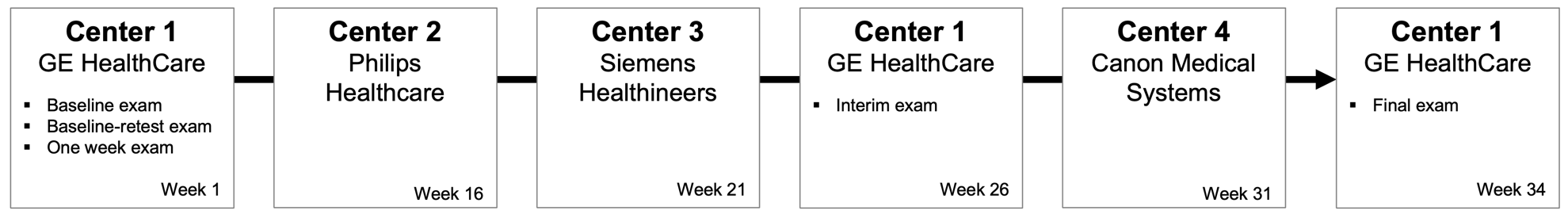


**Supplemental Figure 1:** Phantom shipping workflow and imaging schedule across centers

Imaging at Center 1 was performed at multiple points throughout the study to evaluate phantom integrity and monitor potential drift in proton density fat fraction (PDFF) and T_1_ values. Two exams were conducted during the initial session on the same day (a baseline exam and followed by a retest after repositioning the phantom and reconnecting the coil). Follow-up exams took place at 1 week, 6 months (interim exam; after the phantom was shipped from Center 3 to Center 1) and 9 months (final exam). The phantom was transported between participating centers using an overnight courier service within a protective, foam-padded case.

**
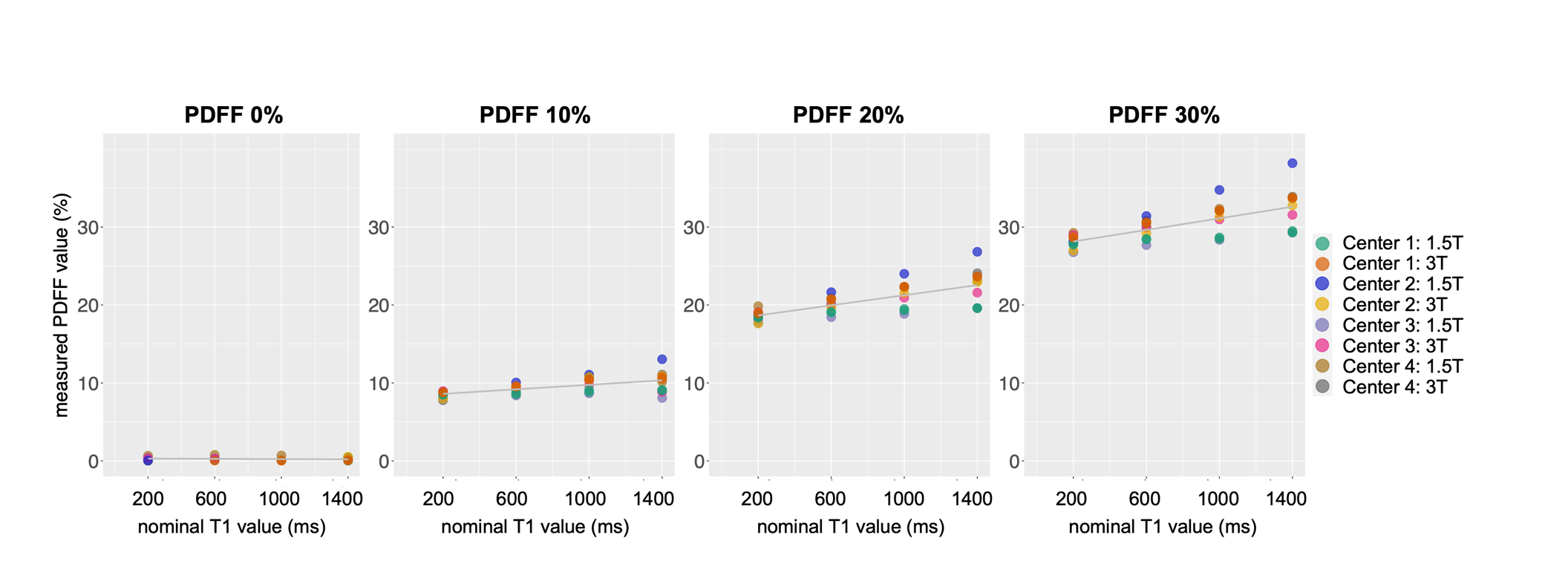
**

**Supplemental Figure 2:** Good reproducibility with low bias was observed for PDFF mapping across PDFF values (0-30%) and T_1_ values (200-1400ms). A moderate overestimation and increased variability were observed in measurements with long T_1_, particularly at higher PDFF values. This effect is likely due to residual T_1_ weighting in conventional PDFF mapping methods. These methods use a low flip angle between 3-5 degrees, which still leads to residual T_1_ bias when the T_1_ of water is much longer than the T_1_ of fat (approximately 350ms).
Notes: PDFF, proton density fat fraction.

**Supplemental Table 1:** Proton density fat fraction (PDFF) and T_1_ reproducibility coefficient (RDC) and percentage RDC (% RDC).

| *Vial No.* | *Vial content* | | **CSE PDFF** Overall RDC: 3.7% | | | **T1 Mapping** Overall RDC: 499ms | | |
| --- | --- | --- | --- | --- | --- | --- | --- | --- |
|  | *PDFF* (%) | *T1w* (ms) | *RDC* (%) | *% RDC* | *% log RDC* | *RDC* (ms) | *% RDC* | *% log RDC* |
| 1 | 0 | 200 | 0.71 | n/a | n/a | 15.6 | 8.59 | 1.62 |
| 2 | 0 | 600 | 0.77 | n/a | n/a | 36.3 | 6.48 | 1.01 |
| 3 | 0 | 1000 | 0.63 | n/a | n/a | 79.1 | 8.42 | 1.22 |
| 4 | 0 | 1400 | 0.63 | n/a | n/a | 161.4 | 11.92 | 1.65 |
| 5 | 10 | 200 | 1.25 | 14.7 | 2.81 | 24.9 | 13.80 | 2.62 |
| 6 | 10 | 600 | 1.44 | 15.6 | 2.45 | 63.6 | 11.06 | 1.72 |
| 7 | 10 | 1000 | 2.43 | 24.1 | 3.59 | 130.4 | 13.29 | 1.91 |
| 8 | 10 | 1400 | 4.34 | 42.4 | 5.82 | 239.1 | 16.97 | 2.38 |
| 9 | 20 | 200 | 2.09 | 11.3 | 2.12 | 21.7 | 12.04 | 2.29 |
| 10 | 20 | 600 | 2.84 | 14.2 | 2.22 | 122.4 | 20.32 | 3.22 |
| 11 | 20 | 1000 | 4.68 | 21.8 | 3.21 | 365.4 | 35.38 | 5.18 |
| 12 | 20 | 1400 | 6.68 | 29.4 | 4.07 | 826.7 | 54.24 | 7.38 |
| 13 | 30 | 200 | 2.48 | 8.8 | 1.67 | 24.2 | 13.36 | 2.51 |
| 14 | 30 | 600 | 3.46 | 11.6 | 1.83 | 250.6 | 40.11 | 6.27 |
| 15 | 30 | 1000 | 5.78 | 18.5 | 2.69 | 748.1 | 67.97 | 9.89 |
| 16 | 30 | 1400 | 7.93 | 24.2 | 3.30 | 1552.5 | 94.54 | 12.50 |
| % RDC calculated as RDC/mean measured value *100  % log RDC calculated based on log-normalized PDFF and T1 values as log RDC/log(nominal value)*100 respectively | | | | | | | | |

**Supplemental Table 2:** Validation of Phantom Homogeneity via SR-CSE T_1_ Mapping.

| **Vial** | **Nominal values** | | **Measured values** | | |
| --- | --- | --- | --- | --- | --- |
|  | **PDFF (%)** | **T1w (ms)** | **Mean T1w (ms)** | **SD** | **CV (%)** |
| 1 | 0 | 200 | 191.1 | 4.6 | 2.43 |
| 2 | 0 | 600 | 606.6 | 2.2 | 0.36 |
| 3 | 0 | 1000 | 1065.6 | 1.3 | 0.12 |
| 4 | 0 | 1400 | 1445.5 | 1.8 | 0.12 |
| 5 | 10 | 200 | 194.7 | 1.5 | 0.76 |
| 6 | 10 | 600 | 613.5 | 1.0 | 0.16 |
| 7 | 10 | 1000 | 1070.2 | 5.2 | 0.48 |
| 8 | 10 | 1400 | 1546.4 | 3.9 | 0.25 |
| 9 | 20 | 200 | 192.1 | 1.1 | 0.59 |
| 10 | 20 | 600 | 615.2 | 2.5 | 0.40 |
| 11 | 20 | 1000 | 1052.6 | 4.5 | 0.43 |
| 12 | 20 | 1400 | 1565.3 | 7.2 | 0.46 |
| 13 | 30 | 200 | 193.4 | 0.4 | 0.20 |
| 14 | 30 | 600 | 612.2 | 1.4 | 0.24 |
| 15 | 30 | 1000 | 1061.5 | 2.4 | 0.22 |
| 16 | 30 | 1400 | 1550.2 | 0.3 | 0.02 |
| PDFF, proton density fat fraction; SD, standard deviation; CV, coefficient of variation | | | | | |
